# Specifying the content of home-based health behaviour change interventions for older people with frailty or at risk of frailty: an exploratory systematic review

**DOI:** 10.1136/bmjopen-2016-014127

**Published:** 2017-02-09

**Authors:** Benjamin Gardner, Ana Jovicic, Celia Belk, Kalpa Kharicha, Steve Iliffe, Jill Manthorpe, Claire Goodman, Vari M Drennan, Kate Walters

**Affiliations:** 1 Department of Psychology, Institute of Psychiatry, Psychology and Neuroscience, King's College London, London, UK; 2 Department of Primary Care and Population Health, University College London, Royal Free Hospital, London, UK; 3 Social Care Workforce Research Unit, King's College London, London, UK; 4 Centre for Research in Primary and Community Care, University of Hertfordshire, Hertfordshire, UK; 5 Centre for Health and Social Care Research, Kingston University & St George's, University of London, London, UK

**Keywords:** Older people, Frailty, Intervention, Behaviour change, Systematic review

## Abstract

**Objectives:**

To identify trials of home-based health behaviour change interventions for frail older people, describe intervention content and explore its potential contribution to intervention effects.

**Design:**

15 bibliographic databases, and reference lists and citations of key papers, were searched for randomised controlled trials of home-based behavioural interventions reporting behavioural or health outcomes.

**Setting:**

Participants' homes.

**Participants:**

Community-dwelling adults aged ≥65 years with frailty or at risk of frailty.

**Primary and secondary outcome measures:**

Trials were coded for effects on thematically clustered behavioural, health and well-being outcomes. Intervention content was described using 96 behaviour change techniques, and 9 functions (eg, education, environmental restructuring).

**Results:**

19 eligible trials reported 22 interventions. Physical functioning was most commonly assessed (19 interventions). Behavioural outcomes were assessed for only 4 interventions. Effectiveness on most outcomes was limited, with at most 50% of interventions showing potential positive effects on behaviour, and 42% on physical functioning. 3 techniques (instruction on how to perform behaviour, adding objects to environment, restructuring physical environment) and 2 functions (education and enablement) were more commonly found in interventions showing potential than those showing no potential to improve physical function. Intervention content was not linked to effectiveness on other outcomes.

**Conclusions:**

Interventions appeared to have greatest impact on physical function where they included behavioural instructions, environmental modification and practical social support. Yet, mechanisms of effects are unclear, because impact on behavioural outcomes has rarely been considered. Moreover, the robustness of our findings is also unclear, because interventions have been poorly reported. Greater engagement with behavioural science is needed when developing and evaluating home-based health interventions.

**PROSPERO registration number:**

ID=CRD42014010370

**Strengths and limitations of this study**

- This is the first systematic review to document the discrete behaviour change content of home-based health behaviour change interventions for frail older people, and explore whether intervention content is related to any potential changes in behavioural, health and well-being outcomes.
- Interventions were coded for their content, using state-of-the-art tools from behavioural science, and for any evidence of potential effectiveness on at least one measure of behaviour, health and/or well-being.
- The main study limitation is that published intervention descriptions lacked detail, such that data entered into the review may be unreliable.
- Nonetheless, our approach points to strategies that may show promise for developers of new home-based health promotion interventions for frail older adults.

## Introduction

Meeting the health and social care needs of an ageing population presents a considerable challenge because of the rising prevalence of frailty, a state of multisystem failure and loss of physiological reserve.^1^ Worldwide, around 11% of people aged 65 or above meet criteria for frailty,^2^ and an estimated 42% have mild frailty or ‘prefrailty’.^1^ Frailty is linked to increased risk of disability, hospital or care home admission, and mortality.^3–6^ Frailty is not inevitable, and may be amenable to intervention.^2^

Many home-delivered interventions designed to reduce functional decline in frail and prefrail populations have focused on behavioural targets, such as dietary change, physical activity and medication adherence.^7–9^ Such interventions can impact positively on health and mortality,^10^ but effects have been mixed: for example, some trials have reported reduced care home admissions,^8^ ^11^ some showed no impact on service use^12^ ^13^ and others noted *increased* long-term service use.^14^ There are two main reasons why such interventions may fail to achieve intended outcomes: behaviour changes do not translate into health and related outcomes, or the behaviour change strategies are ineffective, such that intervention recipients do not modify their behaviour. Past systematic reviews^10^ ^15–17^ have prioritised the former, estimating effectiveness for multiple frailty-related outcomes, though the contribution of particular behaviours (eg, physical activity) to effectiveness was not assessed. To the best of our knowledge, no review has yet described discrete behaviour change strategies or assessed their impact on behavioural and health outcomes. Identifying the ‘active ingredients’ of interventions shifts research emphasis from the question of *how* effective are interventions towards understanding *what determines* their effectiveness.^18^

Identifying intervention components that change behaviour and improve health among frail older adults can guide intervention development. Behavioural science offers tools for categorising and systematically comparing intervention content across studies. A comprehensive taxonomy of behaviour change techniques (BCTs) describes discrete approaches that may be used to potentially change any behaviour (eg, providing information on health consequences, self-monitoring, giving feedback on behaviour^19^), together with nine separate functions that any intervention may serve (eg, education, training, modelling 20). Intervention functions (IFs) represent ‘broad categories of means by which an intervention may change behaviour’ (ref. 20, p. 109), and BCTs the irreducible components that deliver these functions. Applying these frameworks to identify strategies that have been used to change behaviour has three potential benefits. First, it creates a standardised description of intervention methods, enabling replication.^19^ Second, the taxonomy links techniques to theory, so that documenting techniques used in previous interventions may reveal implicit assumptions about the causes of behaviour and behaviour change where explicit theory use is rare.^21^ For example, an intervention offering instructions for healthy eating assumes that poor diet is attributable to lack of knowledge. Finally, comparing techniques and functions in effective versus ineffective interventions can highlight content that may contribute to intervention success.^22^

This review adopts a behavioural science approach to the question: What behaviour change components have been used in home-based health interventions, and how might these components be associated with intervention effectiveness? This review is registered on PROSPERO (CRD42014010370). A published protocol reports finer methodological detail.^23^

## Methods

### Identifying sources for review

#### Eligibility criteria

Included studies met the following PICOS criteria. *Participants* were community-dwelling, aged 65 years or above with, or at risk of, frailty. Those in residential or nursing homes, and hospital inpatients, were excluded. Studies of people aged 50 years or above were eligible where the sample mean age was 65 or above.^11^ Participants were deemed to have or be at risk of frailty were assessed with a validated frailty measure, or considered to be at risk of hospitalisation, or with functional or mobility difficulties, or aged 75 years or above with multiple morbidities. Eligible *interventions* aimed to change health-related behaviours, as delivered in person, solely or primarily within the home, by a health professional, but for which specialist expertise was not required. We included any intervention with a behavioural component, regardless of whether behaviour change was explicitly acknowledged as an intervention target. Studies employing a randomised controlled trial (RCT) design, to *compare* at least one intervention against no treatment or usual care, were included. Studies were eligible where they reported primary quantitative *outcome* data on frailty-relevant behavioural, health or well-being outcomes. Eligible *study types* were peer-reviewed, English full texts published between 1980 and 2014. Single trials reported across multiple sources were treated as single studies.

#### Data sources and search strategy

Two search strategies were used. First, electronic searches were run in September 2014 of 15 health and medical databases: MEDLINE; MEDLINE in Process and Other Non-Indexed Citations; EMBASE; Scopus; Science Citation Index Expanded; Cochrane Database of Systematic Reviews; Cochrane Central Register of Controlled Trials; EPOC; PsycINFO; Health Technology Assessment; National Health Service Economic Evaluation Database; Health Economics Evaluations Database; CINAHL; BiblioMap; and Health Promis. For EPOC and Health Promis, automated search functions were unavailable, so articles were sought via manually searching all publications on the database. For all other databases, an electronic search string specified elder populations, health or behavioural interventions, home settings, and RCT designs, with filters to restrict by date and language (see online supplemental table S1).

10.1136/bmjopen-2016-014127.supp1 supplemental tables

Second, backwards, forward and lateral citation tracking was conducted on records identified via the electronic search where they were systematic reviews retained following abstract screening, or intervention trials retained following full-text screening.

#### Search results and screening

Searches were run by AJ. Two independent researchers (a health psychologist (AJ), and a general practitioner (CB)) screened de-duplicated titles (for obviously irrelevant records), abstracts, and then full texts. Titles and abstracts were rejected where both coders independently deemed them ineligible. Two incidences of coder disagreement over full texts were resolved by senior researchers experienced in ageing and frailty (KK and KW), and behaviour change (BG).

#### Additional materials

Corresponding authors of eligible records were emailed and asked to provide all available additional material. Of 19 authors approached, email addresses were non-functional for 4, 1 had retired and 1 had died. Of 9 who responded, 7 provided additional material, including 14 linked publications (eg, protocols).

### Data extraction

All available material was coded by AJ, with independent second coding (bias risk: CB; all other characteristics: BG) for 6 (32%) records. Coder agreement was assessed, using percentage agreement and κ for all study characteristics combined, each intervention characteristic (behaviour(s) targeted, BCTs, IFs) in isolation, and all outcome data combined. Discrepancies were resolved through discussion.

Study characteristics were extracted for description only*.* Methodological data extracted included country, design, number of arms and interventions, number of follow-up points, time to first follow-up, theory basis, and study-level risk of bias. Theory basis was coded according to whether a named theory of behaviour or behaviour change^24^ was mentioned in the abstract, introduction or method. The Cochrane Collaboration tool was used to assess risk of bias (high, low, unclear) on seven criteria.^25^ Sample characteristics extracted were study eligibility criteria and, within each condition, baseline and follow-up sample size, gender, ethnicity, and health conditions. For one paper in which summary (follow-up) sample sizes were not reported,^12^ the largest recorded total follow-up N was extracted. Reliability for study characteristics was perfect (100% agreement, κ=1^26^).

Intervention characteristics extracted were: behaviour(s) targeted, BCTs and IFs, and delivery methods. We intended to code intervention fidelity, but this was not reported in any paper. Behaviours were coded only where explicitly mentioned; reliability was substantial (79%, κ=0.73). BCTs, identified using an adaptation of the BCT Taxonomy v1,^19^ were coded as absent or, where unequivocal reporting of their administration to at least some intervention recipients, present. Three of 93 BCTs within the taxonomy—practical, emotional and unspecified social support—were each split into two, to differentiate social support from intervention providers versus from friends, family or caregivers. Prior to coding, coders had undertaken (AJ) or administered (BG) BCT coding training (http://www.bct-taxonomy.com). BCT reliability, coded only for techniques deemed present by at least one coder, was almost perfect (90%, κ=0.81). An intervention was coded as performing one or more of nine discrete functions (eg, education, persuasion, training; for definitions, see table 1, footnote).^20^ Function reliability was substantial (88% agreement, κ=0.75). Delivery methods related to who delivered the intervention, in what setting (home only vs home and other), for which reliability was perfect (100%, κ=1). Control treatment characteristics were not extracted because they were rarely reported; 14/19 studies described control treatment only as ‘usual care’.

**Table 1:** Summary of study characteristics (19 studies)

Study characteristics (19 studies)		
Sample size (at first follow-up)		Combined number of participants N=5084 N range 92–477 Median N=254
Study design	RCT	16/19 (84%)
	Cluster RCT	2/19 (11%)
	Pseudo-cluster RCT	1/19 (5%)
Number of arms	2-arm (1 intervention, 1 control)	16/10 (84%)
	3-arm (2 interventions, 1 control)	3/19 (16%)
Time to first follow-up		Range 1 month–2 years Median 6 months
Theory mentioned		3/19 (16%)

RCT, randomised controlled trial.

Outcome data related to all behavioural, health or well-being measures at baseline and first follow-up. P values for mean changes between baseline and follow-up were extracted, with group means and effect sizes, where available. In two trials, p values were taken from trend analyses because outcomes were evaluated only across multiple time points.^27^ ^28^ In one trial,^13^ subgroup analysis (those at least risk of home admission) data were extracted because no other analyses were available. Outcome data reliability was perfect (100% agreement, κ=1).

### Analysis

Two analyses were run. First, discrete components of previous interventions were described. Second, the effectiveness of interventions, and links between components and effectiveness were estimated.

Each extracted outcome variable was inductively classified (by BG) into only one of six mutually exclusive clusters (independently verified by KW): *behavioural* outcomes, representing behaviours or necessarily contingent outcomes (eg, medication adherence, nutritional status); *health and social service use* (eg, hospital admissions); *mental health and functioning* (eg, depression); *physical functioning* (eg, activities of daily living); *social functioning and well-being* (eg, loneliness); and *generic health and well-being* indicators not captured by other clusters (eg, quality of life).

Intervention effectiveness was assessed for each cluster. A dichotomous ‘effectiveness’ variable was created. An intervention was deemed to ‘show evidence of potential effectiveness’ for targeting an outcome where a statistically significant (p<0.05) between-group change in at least one outcome within the cluster favoured the intervention group. Interventions showed ‘no evidence of effectiveness’ where there was no between-group change in any outcome within the cluster, or where statistically significant changes favoured the control group.

The potential contribution to effectiveness of intervention content (ie, behaviour(s) targeted, BCTs, IFs) was assessed by computing an ‘index of potential’ (IP) for each component. This represented, of all interventions in which a component had been used, the percentage found to show ‘evidence of potential effectiveness’. To avoid overinterpreting scant data, indices of potential were calculated only for components in four or more interventions within an outcome cluster. Intervention components were deemed to ‘show potential’ where the IP was above 50%, indicating that the component was present in more effective than ineffective interventions. Components with indices of potential of 50% or less were deemed to show no potential.

## Results

### Description of data set

**Figure 1:**
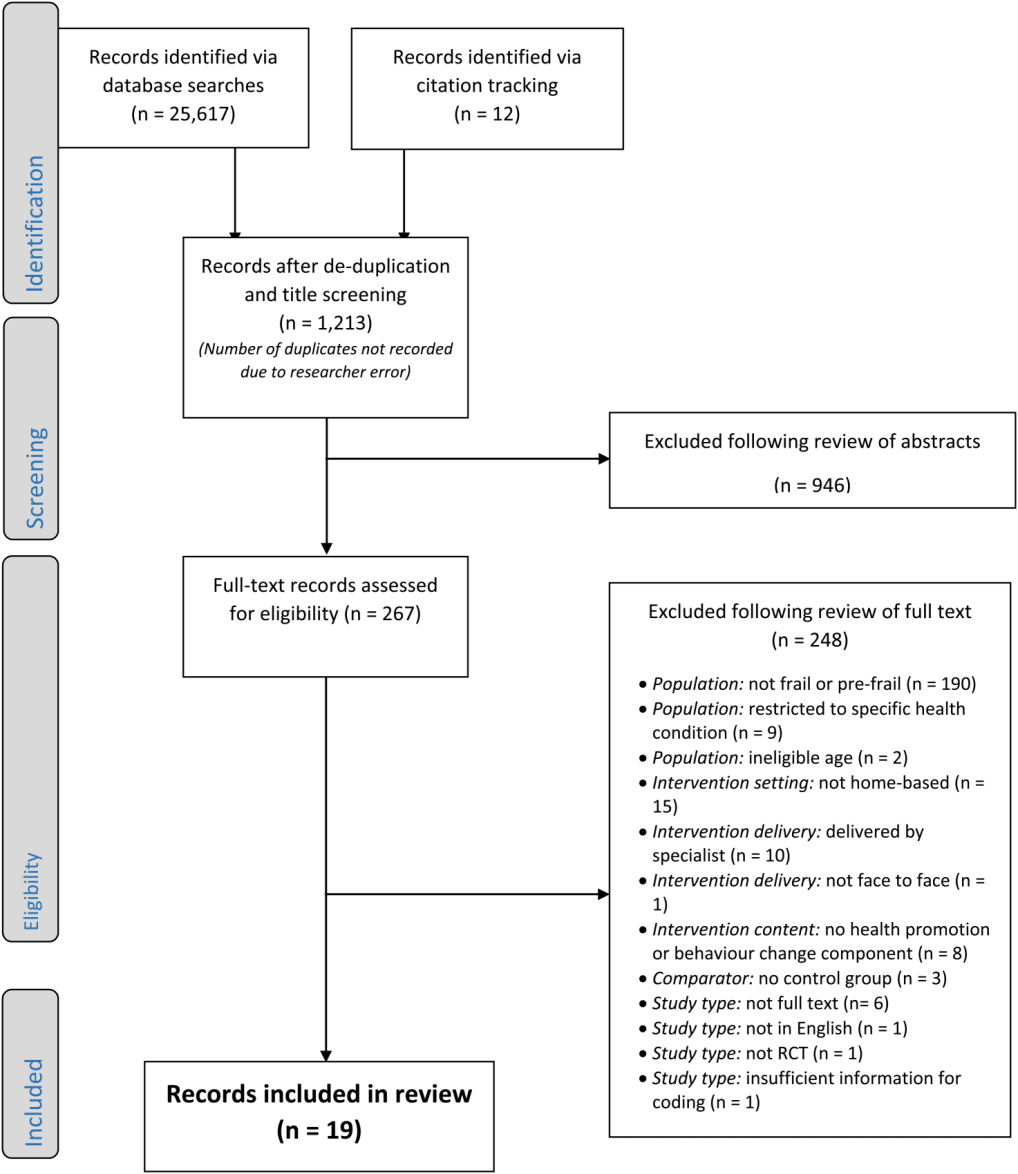
PRISMA flow chart: search strategy and screening procedure. RCT, randomised controlled trial.

Database searches identified 25 617 records, and citation tracking 12 further records. Of these, 24 056 were removed following de-duplication and title screening, 946 following abstract screening, and 248 following full-text screening. The final data set comprised 22 records, reporting 19 trials of 22 eligible interventions (figure 1).

Tables 1 and 2 summarise study and intervention characteristics, and online supplemental table S2 reports further study detail.

**Table 2:** Summary of intervention characteristics (22 interventions)

Intervention characteristics		Number of interventions (total 22 interventions) (%)
Number of behaviours targeted	One behaviour	11 (50%)
	Two behaviours	5 (23%)
	Three behaviours	4 (18%)
	Four behaviours	1 (5%)
	Six behaviours	1 (5%)
Specific behaviours targeted	Alcohol consumption	1 (5%)
	Dietary consumption	8 (36%)
	Medication adherence/management	16 (73%)
	Nutritional supplement intake	1 (5%)
	Physical activity	11 (50%)
	Self-care	1 (5%)
	Sleeping	2 (9%)
	Smoking	2 (9%)
	Vaccination uptake	1 (5%)
Intervention functions*	Education	7 (32%)
	Environmental restructuring	4 (18%)
	Persuasion	2 (9%)
	Training	2 (9%)
	Enablement	16 (73%)
	(No intervention functions identified)	5 (23%)
Setting	Home-only	21 (95%)
	Home and hospital	1 (5%)
Delivered by	Care manager	3 (12%)
	Dietitian	1 (4%)
	Health visitor	1 (4%)
	Home helper	1 (4%)
	Nurse	21 (95%)
	Occupational therapist	4 (16%)
	Physician	1 (4%)
	Physiotherapist	4 (16%)
	Psychologist	1 (4%)
	Social worker	4 (16%)
	Sociologist	1 (4%)
Evidence of potential effectiveness, by outcome cluster	Behavioural (N=4)	Effectiveness: n=2 No effectiveness: n=2
	Health and social service use (N=11)	Effectiveness: n=2 No effectiveness: n=9
	Mental health and functioning (N=11)	Effectiveness: n=3 No effectiveness: n=8
	Physical health and functioning (N=19)	Effectiveness: n=8 No effectiveness: n=11
	Social functioning and well-being (N=7)	Effectiveness: n=1 No effectiveness: n=6
	Generic health and well-being (N=11)	Effectiveness: n=3 No effectiveness: n=8

*Definitions of intervention functions. Education: ‘increasing knowledge or understanding’; environmental restructuring: ‘changing the physical or social context’; persuasion: ‘using communication to induce positive or negative feelings or stimulate action’; training: ‘imparting skills’; enablement: ‘increasing means/reducing barriers to increase capability (beyond education and training) or opportunity (beyond environmental restructuring)’ (ref. 20, p. 7).

Of 19 trials, 9 were undertaken in Europe (4 the Netherlands), 8 in North America (4 USA, 4 Canada), and 1 each in Central America (Mexico) and Australasia (Japan). Sixteen (16/19) trials were individually randomised, 2 cluster randomised and 1 ‘pseudo-cluster’ randomised, whereby those delivering the intervention were randomised to conditions determining participant randomisation ratios. Sixteen trials used two-arm designs and three three-arm designs, all of which evaluated two interventions. Time to first follow-up ranged from 1 to 36 months (median 6 months).

Behaviour change theories were mentioned in only 3 (3/19) trials. All trials showed low bias risk on at least four of seven criteria, with three trials at low risk on all criteria (see online supplemental table S3).

In total, data for 5084 participants were available at first follow-up (N range 92–477; median N=254). Interventions most commonly targeted one behaviour (11/22 interventions). The most commonly targeted behaviours were medication adherence or management (16/22), and physical activity (11/22). Most (21/22) interventions were delivered solely within the home, and 2 in home and hospital settings. Most (21/22) were delivered by nurses.

Of the 96 possible BCTs, 21 were each identified in at least 1 intervention (see online supplementary table S4). Number of BCTs per intervention ranged from 1 to 9 (median 4.5; mean 4.4; mode 5). The most frequently used were monitoring of behaviour by others without feedback, and practical and unspecified social support from intervention providers (each in 13/22 interventions).

Five functions were each coded in at least one intervention. The number of functions per intervention ranged from 1 to 3 (median 2; mean 1.5; mode 2), though functions could not be coded for five interventions. Common functions were enablement (16/22 interventions) and education (7/22).

### Comparing effective and ineffective interventions

Of the 19 interventions assessed on physical health and functioning outcomes, 8 showed evidence of potential effectiveness (tables 3 and 4). Potential effectiveness was shown for: 2 of 4 interventions assessed on behavioural outcomes; 2 of 11 on health and social service use; 3 of 11 on mental health and functioning; 1 of 7 on social functioning and well-being; and 3 of 11 on generic health and well-being.

**Table 3:** Intervention effectiveness in the outcome clusters physical functioning, behavioural outcomes, and health and social service use according to behaviour targeted, intervention functions and behaviour change techniques*

Physical functioning outcomes				
	Evidence of potential effectiveness (κ=8)	No evidence of effectiveness (κ=11)	All (κ=19)	Index of potential†
Behaviours targeted				
Dietary consumption	3	4	7	43%
Medication adherence/management	5	8	13	38%
Physical activity	3	7	11	27%
Intervention functions				
Education	**5**	**1**	**6**	**83%**
Enablement	**7**	**6**	**13**	**54%**
Environmental restructuring	2	3	5	40%
(None identified)	1	4	5	−
Behaviour change techniques				
Adding objects to the environment	**3**	**2**	**5**	**60%**
Goal setting (outcome)	4	5	9	44%
Instruction on how to perform behaviour	3	1	4	75%
Monitoring of behaviour by others without feedback	2	2	4	50%
Monitoring of outcomes of behaviour by others without feedback	**3**	**9**	**12**	**25%**
Restructuring the physical environment	3	2	5	**60%**
Social support from intervention provider (practical)	5	5	10	50%
Social support from intervention provider (unspecified)	4	7	11	36%
**Behavioural outcomes**				
	**Evidence of potential effectiveness (κ=2)**	**No evidence of effectiveness (κ=2)**	**All (κ=4)**	**Index of potential†**
Behaviour change techniques				
Monitoring of outcomes of behaviour by others without feedback	2	2	4	50%
**Health and social service use outcomes**				
	**Evidence of potential effectiveness (κ=2)**	**No evidence of effectiveness (κ=9)**	**All (κ=11)**	**Index of potential**†
Behaviours targeted				
Dietary consumption	1	3	4	25%
Medication adherence/management	2	7	9	22%
Physical activity	1	4	5	20%
Intervention functions				
Enablement	2	4	6	33%
Behaviour change techniques				
Monitoring of outcomes of behaviour by others without feedback	1	9	10	10%
Social support from intervention provider (practical)	2	3	5	40%
Social support from intervention provider (unspecified)	2	7	9	22%

*Only characteristics identified in at least four interventions within each cluster are reported for that cluster.

†‘Index of potential’ refers to the percentage of studies, of all those featuring the focal intervention characteristic, found to show evidence of potential effectiveness on at least one variable within the relevant outcome cluster. Rows in bold denote components found to show promise (index of potential >50%).

**Table 4:** Intervention effectiveness in the outcome clusters mental health and functioning, social functioning/well-being, and generic health and well-being according to behaviour targeted, intervention functions and behaviour change techniques*

Mental health and functioning outcomes				
	Evidence of potential effectiveness (κ=3)	No evidence of effectiveness (κ=8)	All (κ=11)	Index of potential†
Behaviours targeted				
Medication adherence/management	2	5	7	29%
Intervention functions				
Enablement	2	3	5	40%
Environmental restructuring	1	3	4	25%
(None identified)	1	5	6	−
Behaviour change techniques				
Goal setting (outcome)	3	3	6	50%
Monitoring of outcomes of behaviour by others without feedback	2	7	9	22%
Social support from intervention provider (practical)	2	2	4	50%
Social support from intervention provider (unspecified)	2	5	7	29%
**Social functioning and well-being outcomes**				
	**Evidence of potential effectiveness (κ=1)**	**No evidence of effectiveness (n=6)**	**All (κ=7)**	**Index of potential†**
Behaviours targeted				
Physical activity	0	4	4	0%
Intervention functions				
(None identified)	0	3	3	−
Behaviour change techniques				
Goal setting (outcome)	1	4	5	20%
Monitoring of outcomes of behaviour by others without feedback	1	6	7	14%
Social support from intervention provider (unspecified)	1	5	6	17%
**Generic health and well-being outcomes**				
	**Evidence of potential effectiveness (κ=3)**	**No evidence of effectiveness (κ=8)**	**All (κ=11)**	**Index of potential**†
Behaviours targeted				
Dietary consumption	2	3	5	40%
Medication adherence/management	3	5	8	38%
Physical activity	2	3	5	40%
Intervention functions				
Enablement	3	6	9	33%
Behaviour change techniques				
Goal setting (outcome)	1	3	4	25%
Monitoring of outcomes of behaviour by others without feedback	0	6	6	0%
Social support from intervention provider (practical)	3	3	6	50%
Social support from intervention provider (unspecified)	0	7	7	0%

*Only characteristics identified in at least four interventions within each cluster are reported for that cluster.

†‘Index of potential’ refers to the percentage of studies, of all those featuring the focal intervention characteristic, found to show evidence of potential effectiveness on at least one variable within the relevant outcome cluster. Rows in bold denote components found to show promise (index of potential >50%).

Three BCTs and two functions showed potential for improving physical functioning outcomes (table 3). The BCTs were: providing instruction on how to perform the behaviour (eg, how to use medication;^9^ IP=75%); adding objects to the environment (eg, medication dispenser;^27^ IP=60%) and restructuring the physical environment (eg, making housing modifications to reduce fall risks;^9^ IP=60%). Functions showing potential were education (IP=86%) and enablement (IP=53%).

No single BCT or function showed potential for modifying outcomes relating to behaviour, health and social service use (table 3), mental health and functioning, social functioning and well-being, or generic health and well-being (table 4). The behaviours targeted showed no potential for any outcome.

## Discussion

Twenty-two home-delivered health behaviour change interventions for older people who are frail or at risk of frailty showed mixed effects on behavioural, health or well-being outcomes: 8 of 11 showed potential to improve physical function, 2 of 4 interventions changed behaviour and no more than 27% of interventions showed any potential to improve mental health and functioning, social functioning and well-being, or generic health and well-being, or reduce service use. Three BCTs and two IFs were more common in interventions showing potential to enhance physical functioning, though no components were consistently linked to other outcome types. Use of behavioural science in developing and evaluating these interventions appeared limited: behavioural outcomes were rarely assessed, explicit theory use scant and intervention components poorly reported. Nonetheless, findings offer tentative guidance to intervention developers as to which components most warrant further investigation in future home-based health promotion initiatives.

Our definition of interventions as ‘showing potential’ where positive effects were found in at least one measure of a given outcome may have overestimated effectiveness. For example, one intervention ‘showing potential’ changed only two of four health and social service use indicators.^11^ Additionally, we coded but did not weight results for risk of bias. These crude analysis techniques were used to identify interventions and components showing any potential, however small or potentially biased, to improve behaviour, health or well-being. Consequently, the lack of effects observed in trials where multiple outcome measures were employed is notable, as it indicates a comprehensive absence of effects.^12^ ^28–30^ On the other hand, our effectiveness estimates were conservative, based on changes at first follow-up, irrespective of whether studies were powered to detect changes. Some interventions had effects only at later follow-up.^31^ In the absence of a common follow-up duration across all trials however, we anticipated that most change would be observed at initial follow-up, with behaviour and health gains dissipating over time, which is the typical change trajectory for behavioural interventions.^32^

We applied state-of-the-art coding technologies to describe interventions, but coding validity depends on the clarity of intervention descriptions, which we found to be poorly specified. This may in part be because many of the interventions reviewed were not conceived by their authors as behavioural interventions, such as case-management strategies targeting aimed at modifying the behaviour or organisation of professionals involved in care provision.^12^ ^31^ ^33^ However, all interventions sought to modify health-related behaviours of frail older people, which may potentially have contributed to effectiveness. While intercoder reliability was good, our findings are based on our post hoc interpretations of intervention content, rather than comprehensive reports of true content written by intervention developers. Consequently, several interventions could not be coded for functions. Additionally, intervention effectiveness is partly dependent on the control conditions against which it has been compared;^34^ techniques that are present in intervention and control arms cannot be said to independently contribute to intervention effectiveness. Yet, we were unable to code the content of control treatments due to lack of information, so our analysis will have overestimated the potential of any technique that was included in the intervention and control arms.^34^ Intervention developers must describe carefully the behavioural components of intervention and control treatments to permit more accurate evidence syntheses. The BCT Taxonomy v1 and IF list are useful for standardising description.^19^ ^20^ We estimated the contribution of intervention components to potential effects through comparing interventions yielding statistically significant effects with those with no effects. Notwithstanding the lack of information on comparison treatments for assessing effectiveness at the within-study level, more sophisticated methods are available for quantifying relationships between content and between-study variation in effectiveness.^35^ Yet, powering such analyses requires larger sample sizes and the validity of results with more homogeneous outcomes, than were available. Our analysis assumed that BCTs represent the ‘active ingredients’ of interventions,^19^ so focused on intervention content, but effects may depend on complex interactions between content, delivery, format and setting.^36^ More fundamentally, our analysis focused on behavioural elements of interventions that were in many cases multifaceted, such that modifying the behaviour of frail older people was only one of the multiple strategies employed to improve health. Nonetheless, our findings offer a step towards documenting the behaviour-related content of home-based health behaviour change interventions for prefrail and frail older people, highlighting content that appears to show promise, across contexts, for improving health.

Interventions most commonly sought to promote health by targeting improved medication management, greater physical activity or a healthier diet, and effects were assessed against six types of behavioural, health and well-being outcomes. Such diverse content demonstrates the importance of asking *whether* interventions are effective, and *what makes* them effective, and against which outcomes.^18^ Physical functioning outcomes were most frequently assessed. Interventions showing potential for improving physical functioning were more likely to seek to educate frail older people in why and how to make behavioural changes, or to increase their capability or opportunity for change. These interventions more frequently included techniques based on instructions on how to perform recommended behaviours, and environmental modifications to support change (eg, removing physical obstacles in the home to permit walking^9^). Given the methodological limitations of our review, we cannot conclude that these techniques are uniformly effective for improving physical functioning among frail older adults. All three techniques were present in both interventions that showed evidence of potential effectiveness for improving physical functioning and those that did not. Moreover, 75 of 96 possible techniques were not used in any intervention, so their potential for changing behaviour and health of frail older people cannot be ruled out. Nonetheless, given the centrality of physical functioning as a frailty marker,^3^ future interventions should consider adopting these strategies. Some studies within this review excluded those likely to be the most frail (eg, those with severe dementia, or receiving home nursing services) and we excluded studies based exclusively within nursing or care home settings, and our findings may not apply to these populations.

Surprisingly, across most outcomes, most of the components that we identified were more commonly found in interventions that had *no* impact. For example, monitoring outcomes of participants' behaviour without providing feedback (eg, assessing but not informing participants of their physical health^7^) was more consistently found in interventions with no effect on mental, physical or generic health indices, nor social functioning. This need not mean these techniques are inherently less effective for health promotion among frail older people. Notably few trials assessed effectiveness against behavioural outcomes. Consequently, it is unclear whether components prompted behaviour changes that did not yield health benefits, or failed to prompt behaviour change. Evaluating change only in health and related outcomes (eg, number of falls^29^ ^37^), rather than behaviour that may prompt such changes (eg, physical activity^38^), limits understanding of reasons for intervention effects, or lack thereof. Behaviour change interventions should be evaluated against behavioural criteria in addition to important outcomes for frail older people, such as functional ability.

Changing behaviour requires understanding of the determinants of behaviour.^39^ Yet, only 3 of 22 interventions were explicitly based on theories of behaviour change. Behaviour change theories provide hypotheses around the processes that generate behaviour, and offer targets for behavioural interventions.^39^ For example, the‘COM-B’ model proposes that behaviour (B) is determined by capability (C), opportunity (O) and motivation (M).^20^ In applying this model, for those who are already sufficiently motivated—for example, an underweight frail older person is motivated to eat a more calorie-dense, protein-rich diet—behaviour change thus depends on enhancing perceptions of capability and opportunity to act, for example, in this instance buying and preparing suitable food. In the absence of explicit theory use, commonly employed techniques and IFs can reveal implicit theoretical assumptions underpinning interventions. The most commonly used BCTs were monitoring of behaviour without feedback, and practical or unspecified social support from the intervention provider, and common functions were enablement and education. These strategies indicate that intervention developers have implicitly touched on all three COM-B domains, conceiving of health promotion among frail older people as dependent on enhancing motivation via education about the importance of health behaviour, and targeting capability and opportunity via social support to enable behaviour change. Nonetheless, we encourage developers to articulate and assess the theoretical mechanisms through which health promotion is expected to impact on health, thereby improving understanding of how interventions take effect. Practical guidance is available for moving from assumptions about what needs to change, to selection of theory-based intervention methods.^39^ ^40^

Interventions that instruct and inform frail older people in how and why to change their behaviour, or support physical environment modifications, appear to show promise for improving physical function. Yet, the robustness of these findings is unclear. Some components were identified in few interventions. Any potentially eligible study published since we conducted our review^41–43^ that used these components may alter relationships with potential effectiveness. Moreover, it is premature to form conclusions about what makes effective home-based health behaviour change interventions, because behaviour change is rarely assessed, and intervention content poorly reported. Developers should engage with behavioural science in designing, evaluating and reporting interventions.
