# Supplementary material for: Specifying the content of home-based health behaviour change interventions for older people with frailty or at risk of frailty: an exploratory systematic review
Source: BMJ Open. 2017 Feb 9;7(2):e014127. doi: 10.1136/bmjopen-2016-014127 (PMC5306507; doi:10.1136/bmjopen-2016-014127)
Supplement: supplemental tables [file bmjopen-2016-014127supp_tables.pdf]

Supplemental Table 1. Sample search string, used on Ovid MEDLINE

**Step**	**Search term**
1. (Population)	(“old* age” OR “aging” OR “ageing” OR “old* adult*” OR “old* people” OR “elder*” OR “geriatric*” OR “senior*” OR “pensioner*” OR “over 65” OR “over sixty five” OR “over sixty-five” OR “65+” OR “veteran*” OR “frail*”).mp
2. (Intervention)	(“health promotion*” OR “behavio* chang*” OR “healthy aging” OR “healthy ageing” OR “health education” OR “intervention*” OR “lifestyle*” OR “wellbeing” OR “health campaign*” OR “health prevent*” OR “health protect*” OR “primary prevent*” OR “case manag*” OR “diet*” OR “nutrition” OR “healthy eating” OR “exercis*” OR “physical activit*” OR “alcohol” OR “smok*” OR “mood*” OR “depress*” OR “anxi*” OR “psycholog*” OR “cogniti*” OR “fall* prevent*” OR “polypharmacy” OR “prevent* hospital*”).mp
3. (Setting)	(“Home-based” OR “homebased” OR “house-based” OR “housebased” OR “community-dwelling” OR “community dwelling” OR “domiciliary” OR “outreach” OR “home”).mp
4. (Study type)	(“Trial” OR “randomi* control*” OR “RCT”).mp
5.	1 AND 2 AND 3 AND 4
6. (Limits)	Limit 5 to (English language and full text and humans and yr=“1980-2014”)

Supplemental Table 2. Description of studies

Study characteristics	*Description of intervention and control treatment(s)*	*Sample characteristics*			*Intervention characteristics*	*Outcomes measured*	*Summary of findings*
		Sample description	*Intervention group(s) description*	*Control group description*			
Avlund et al [1] (also Vass et al) [2]<br><br>Denmark<br><br>RCT, 2 arms (1 intervention,	Intervention: Health assessment and development of tailored plan<br><br>Control: Usual care	Aged 60+y (geriatric wards) or 70+y (medical wards), requiring ongoing treatment and home services	Baseline N = 59<br><br>Follow-up N = 57 **<br><br>Mean age (estimated) ≥	Baseline N = 90<br><br>Follow-up N = 82<br><br>Mean age (estimated):	Behaviours targeted: Dietary consumption, medication use, PA<br><br>BCTs: Monitoring of outcomes by others without feedback, social support from	*Health and social service use*<br>Hospital admissions<br><br>*Physical functioning*	*Health and social service use:*<br>**No evidence of effectiveness**<br>****<br><br>*Physical functioning:*

1 control)<br><br>Number of follow-up points: 1<br><br>Follow-up: 3 months<br><br>Low risk of bias 5/7<br><br>No theory mentioned		Excluded: impressive aphasia, severe dementia, terminal illness, addiction problems, or hospitalized <4 days	67y ***<br><br>Gender NR<br><br>Ethnicity NR<br><br>Health conditions NR	67y<br><br>Gender NR<br><br>Ethnicity NR<br><br>Health conditions NR	intervention provider (practical), social support from intervention provider (unspecified)<br><br>Functions: Enablement, persuasion<br><br>Setting: Home-only<br><br>Delivered by: Home nurse, home helper, physiotherapist, or occupational therapist (according to individual	Functional performance	**No evidence of effectiveness**

					older person's needs)		
Boult et al [3] (also Boyd et al [4])<br><br>USA<br><br>Cluster RCT, 2 arms (1 intervention, 1 control)<br><br>Number of follow-up points: 1	<u>Intervention:</u> Primary-care based care management, transitional care, and support for self-management and family caregiving<br><br><u>Control:</u> Usual care	Aged 65+y, eligible for Medicare or TriCare insurance, at high risk of generating high health care expenditure in coming year	Baseline N = 485<br><br>Follow-up N = 274<br><br>Mean age: 77y<br><br>54% female<br><br>51% Caucasian, 46% African-American	Baseline N = 419<br><br>Follow-up N = 203<br><br>Mean age: 78y<br><br>55% female<br><br>49% Caucasian, 46% African-American	Behaviours targeted: PA, diet, sleeping, medication use, smoking, alcohol consumption<br><br>BCTs: Discrepancy between current behaviour and goal, monitoring behaviour without feedback, self-monitoring (outcome), social support from intervention provider (practical), social	*Health and social service use*<br>Health service use<br><br>*Physical functioning*<br>Functional performance<br><br>*Generic health and wellbeing*<br>Mortality	*Health and social service use*<br>**Evidence of potential effectiveness**<br>Reduction in home health care episodes<br><br>*Physical functioning*<br>**No evidence of effectiveness**<br><br>*Generic health*

Follow-up: 3 years<br><br>Low risk of bias 7/7<br><br>Theory mentioned (Transtheoretical Model)			Mean number of health conditions 4.3	Mean number of health conditions 4.3	support from intervention provider (unspecified)<br><br>Functions: Enablement, persuasion<br><br>Setting: Home-only<br><br>Delivered by: Nurse		*and wellbeing*<br>**No evidence of effectiveness**
Bouman et al [5] (also Nicolaides-Bouman et al [6])	<u>Intervention:</u> Assessment of health problems or risks, provision of advice, and	Aged 70-84y, living at home<br><br>Excluded: Receiving regular home	Baseline N = 160<br><br>Follow-up N = 115	Baseline N = 170<br><br>Follow-up N = 139	Behaviours targeted: Dietary consumption, PA<br><br>BCTs: Goal setting (outcome), monitoring	*Physical functioning*<br>Functional status<br>ADLs<br>IADLs	*Physical functioning*<br>**No evidence of effectiveness**<br><br>*Social functioning*

Netherlands<br><br>RCT, 2 arms (1 intervention, 1 control)<br><br>Number of follow-up points: 3<br><br>First follow-up: 12 months<br><br>Low risk of bias 6/7	referral to other services<br><br><u>Control:</u> Usual care	care	Age range: 70-84y<br><br>60% female<br><br>Ethnicity NR<br><br>Health conditions NR	Age range: 70-84y<br><br>60% female<br><br>Ethnicity NR<br><br>Health conditions NR	of outcomes by others without feedback, review behavioural goals, social support from intervention provider (unspecified)<br><br>Functions: Enablement<br><br>Setting: Home-only<br><br>Delivered by: Home nurses	*Social functioning and wellbeing*<br>Social support<br>Loneliness<br><br>*Generic health and wellbeing*<br>General health<br>Quality of life	*and wellbeing*<br>**No evidence of effectiveness**<br><br>*Generic health and wellbeing*<br>**No evidence of effectiveness**

No theory mentioned							
Dalby et al [7]<br><br>Canada<br><br>RCT, 2 arms (1 intervention, 1 control)<br><br>Number of follow-ups: 1<br><br>Follow-up: 14 months	Intervention:<br>Assessment of health and wellbeing problems and risks, and development of personalized care plan<br><br>Control:<br>Usual care	70+y, with functional impairment, admission to hospital, or bereavement in previous 6 months<br><br>Excluded:<br>Living in nursing home, or had previous nurse home	Baseline N = 73<br><br>Follow-up N = 59<br><br>Mean age: 79y<br><br>71% female<br><br>Ethnicity NR<br><br>Three most prevalent	Baseline N = 69<br><br>Follow-up N = 54<br><br>Mean age: 78y<br><br>62% female<br><br>Ethnicity NR<br><br>Three most prevalent	Behaviours targeted:<br>Medication adherence, vaccination (influenza and pneumonia)<br><br>BCTs: Monitoring of behaviour by others without feedback, monitoring of outcomes of behaviour by others without feedback, social support from friends/family/caregivers (unspecified), social	*Behavioural*<br>Influenza and pneumonia vaccination rate<br><br>*Health and social service use*<br>Institutional admissions<br>Health service use<br><br>*Generic health*	*Behavioural*<br>**Evidence of potential effectiveness**<br><br>*Health and social service use*<br>**No evidence of effectiveness**<br><br>*Generic health and wellbeing*<br>**No evidence of effectiveness**

Low risk of bias 5/7<br><br>No theory mentioned		visits	health conditions: arthritis (51%), hypertension (37%), heart condition (30%)	health conditions: arthritis (51%), hypertension (35%), heart condition (28%)	support from intervention provider (practical), social support from intervention provider (unspecified)<br><br>Functions: Enablement<br><br>Setting: Home-only<br><br>Delivered by: Primary care nurse	*and wellbeing*<br>Mortality	
Favela et al [8]<br><br>Mexico	<u>Intervention 1:</u> Assessment of health and	70-90y, eligible for national medical	<u>Intervention 1 (alert button):</u>	Baseline N = 44	<u>Intervention 1:</u> Behaviours targeted: PA, medication	*Physical functioning*<br>Frailty	<u>Intervention 1 (alert button)</u><br>*Physical*

RCT, 3 arms (2 intervention, 1 control)<br><br>Number of follow-ups: 1<br><br>Follow-up: 9 months<br><br>Low risk of bias 4/7<br><br>No theory mentioned	development of health improvement plan, with alert button to summon emergency care<br><br><u>Intervention 2:</u> Assessment of health and development of health improvement plan	insurance<br><br>Excluded: N/A	Baseline N = 45<br><br>Follow-up N = 39<br><br>Age range: 70-90y<br><br>40% male<br><br>Ethnicity NR<br><br>Prevalence of health conditions:	Follow-up N = 39<br><br>Age range: 70-90y<br><br>48% male<br><br>Ethnicity NR<br><br>Prevalence of health conditions: cognitive impairment (30%),	adherence<br><br>BCTs: Action planning, adding objects to the environment, goal setting (outcome), graded tasks, instruction on how to perform behaviour, monitoring of behaviour by others without feedback, review outcome goals, social support from friends/family/caregivers (unspecified), social		*functioning*<br>**Evidence of potential effectiveness**<br><br><u>Intervention 2 (no alert button)</u><br>*Physical functioning*<br>**No evidence of effectiveness**

	Control:<br>Usual care		depression (30%), cognitive impairment (23%)<br><br>Intervention 2 (no alert button):<br><br>Baseline N = 44<br><br>Follow-up N = 37	depression (23%)	support from intervention provider (practical)<br><br>Functions: Enablement, training<br><br>Setting: Home-only<br><br>Delivered by: Nurse<br><br>Intervention 2:<br>Behaviours targeted: PA, medication adherence		

			Age range: 70-90y<br><br>48% male<br><br>Ethnicity NR<br><br>Prevalence of health conditions: depression (33%), cognitive impairment (33%)		BCTs: Action planning, goal setting (outcome), graded tasks, instruction on how to perform behaviour, monitoring of behaviour by others without feedback, review outcome goals, social support from friends/family/caregivers (unspecified), social support from intervention provider (practical)		

					Functions: Enablement, training<br><br>Setting: Home-only<br><br>Delivered by: Nurse		
Gustafsson et al [9]<br>(also Behm, Dahlin-Ivanoff & Zidén [10]; Behm, Wilhelmson et al [11]; Behm, Zidén et al [12]; Dahlin-	<u>Intervention 1 (home visit only):</u><br>Provision of advice on available support services<br><br><u>Intervention 2</u>	Aged 80+y, living at home<br><br>Excluded: dependent on home help service or care, receiving help for ADLs, or overt cognitive	<u>Intervention 1 (home visit only)</u><br><br>Baseline N = 174<br><br>Follow-up N = 157	Baseline N = 114<br><br>Follow-up N = 88<br><br>Age range: 80-97y<br><br>61% female	<u>Intervention 1 (home visits only):</u><br>Behaviours targeted: PA, medication use, diet<br><br>BCTs: Instruction on how to perform behaviour, restructuring physical environment,	*Physical functioning*<br>Frailty<br>ADLs<br><br>*Generic health and wellbeing*<br>Symptoms<br>General health	<u>Intervention 1:</u><br>*Physical functioning*<br>**Evidence of potential effectiveness**<br>Less dependence in ADLs<br><br>*Generic health*

Ivanoff et al [13])<br><br>Sweden<br><br>RCT, 3 arms (2 interventions, 1 control)<br><br>Number of follow-up points: 3<br><br>First follow-up: 3 months	<u>(senior meeting * home visit):</u> Multidisciplinary discussions, followed by provision of advice on available support services<br><br><u>Control:</u> Usual care	impairment	Age range: 80-94y<br><br>64% female<br><br>Ethnicity NR<br><br>Health conditions NR<br><br><u>Intervention 2 (senior meetings * home visit)</u><br><br>Baseline N =	Ethnicity NR<br><br>Health conditions NR	social support from intervention provider (practical)<br><br>Functions: Education, enablement<br><br>Setting: Home-only<br><br>Delivered by: Occupational therapist, physiotherapist, nurse, or social worker<br><br><u>Intervention 2 (senior meetings * home visit):</u>		*and wellbeing* **Evidence of potential effectiveness** Less general deterioration of health<br><br><u>Intervention 2:</u> *Physical functioning* **Evidence of potential effectiveness** Less dependence in ADLs

Low risk of bias 6/7<br><br>No theory mentioned			171<br><br>Follow-up N = 147<br><br>Age range: 80-94y<br><br>66% female<br><br>Ethnicity NR<br><br>Health conditions NR		Behaviours targeted: PA, medication use, diet<br><br>BCTs: Information on health consequences, instruction on how to perform behaviour, restructuring physical environment, social support from intervention provider (practical)<br><br>Functions: Education, enablement		*Generic health and wellbeing*<br>**Evidence of potential effectiveness**<br>Less deterioration of general health,

					Setting: Home-only<br><br>Delivered by: Occupational therapist, physiotherapist, nurse, or social worker		
Hall et al [14]<br><br>Canada<br><br>RCT, 2 arms (1 intervention, 1 control)<br>******	<u>Intervention:</u> Standard personal care at home, with development of personal health plan<br><br><u>Controls:</u>	Aged 65+y, living at home, newly admitted to receive personal home-care<br><br>Excluded: N/A	Baseline N = 81<br><br>Follow-up N = 81<br><br>Mean age: 78y<br><br>79% female	Baseline N = 81<br><br>Follow-up N = 81<br><br>Mean age: 78y<br><br>68% female	Behaviours targeted: Dietary consumption, medication over-use, PA, smoking<br><br>BCTs: Goal setting (outcome), monitoring of outcome of behaviour by others	*Health and social service use*<br>Uptake of more intensive support services<br>Institutional admissions	*Health and social service use*<br>**No evidence of effectiveness**<br><br>*Generic health and wellbeing*<br>**No evidence of effectiveness**

Number of follow-ups: 3<br><br>First follow-up: 12 months<br><br>Low risk of bias 6/7<br><br>No theory mentioned	Standard personal care at home		Ethnicity NR<br><br>Three most prevalent health conditions: 42% had heart disease, 35% had high blood pressure, 62% had arthritis	Ethnicity NR<br><br>Three most prevalent health conditions: 44% had heart disease, 32% had high blood pressure, 46% had arthritis	without feedback, review outcome goals, social support from intervention provider (emotional), social support from intervention provider (unspecified)<br><br>Functions: Enablement<br><br>Setting: Home-only<br><br>Delivered by: Nurse	*Generic health and wellbeing*<br>Mortality	
Kono et al [15] (also Kono et	<u>Intervention:</u> Assessment of	Aged 65+y, living at home,	Baseline N = 161	Baseline N = 162	Behaviour targeted: PA	*Physical functioning*	*Physical functioning*

al [16])<br><br>Japan<br><br>RCT, 2 arms (1 intervention, 1 control)<br><br>Number of follow-ups: 2<br><br>First follow-up: 12 months<br><br>Low risk of bias 5/7	health or psychosocial problems and development of personalized recommendations<br><br><u>Control:</u><br>Usual care	requiring long-term care<br><br>Excluded: Have used formal long-term care services in past 3 months	Follow-up N = 132<br><br>Mean age: 80y<br><br>74% female<br><br>Ethnicity NR<br><br>Health conditions NR	Follow-up N = 127<br><br>Mean age: 80y<br><br>74% female<br><br>Ethnicity<br><br>Health conditions NR	BCTs: Monitoring of behaviour by others without feedback, monitoring of outcomes by others without feedback, social support from family/friends/caregiver (unspecified), social support from intervention provider (practical)<br><br>Functions: (None identified)	ADLs<br>IADLs<br><br>*Health and social service use*<br>Long-term home care use<br><br>*Mental health and functioning*<br>Depression<br><br>*Social functioning and wellbeing*	**No evidence of effectiveness**<br><br>*Health and social service use*<br>**No evidence of effectiveness**<br>*Increased* long-term service use<br><br>*Mental health and functioning*<br>**No evidence of effectiveness**<br><br>*Social functioning*

No theory mentioned					Setting: Home-only<br><br>Delivered by: Community health nurse, care manager, or social worker	Social support	*and wellbeing*<br>**No evidence of effectiveness**
Levine et al [17]<br><br>USA<br><br>RCT, 2 arms (1 intervention, 1 control)<br><br>Number of	<u>Intervention:</u> Assessment of health problems, health education, advice on disease management, and care	Frail, at high risk for use of medical services<br><br>Unclear whether age an eligibility criterion<br><br>Excluded: N/A	Baseline N = 156<br><br>Follow-up N = Unclear (total sample N = 253)<br><br>Mean age: 81y	Baseline N = 142<br><br>Follow-up N = Unclear (total sample N = 253)<br><br>Mean age: 81y	Behaviour targeted: Medication adherence<br><br>BCTs: Monitoring of outcomes by others without feedback, social support from intervention provider (practical), social support from	*Health and social service use*<br>Inpatient service use<br>Emergency dept admission<br>Visits to physician<br>Health service	*Health and social service use*<br>**Evidence of potential effectiveness**<br>Less inpatient service use, fewer visits to physician

follow-ups: 1<br><br>Follow-up: 6 months<br><br>Low risk of bias 6/7<br><br>No theory mentioned	planning<br><br><u>Control:</u><br>Usual care		70% female<br><br>60% White, 12% Black, 21% non-White Hispanic<br><br>Three most prevalent health conditions: renal failure (55%), diabetes (52%),	64% female<br><br>63% White, 12% Black, 15% non-white Hispanic<br><br>Three most prevalent health conditions: renal failure (61%), diabetes (53%), congestive	intervention provider (unspecified)<br><br>Functions: Education, enablement<br><br>Setting: Home-only<br><br>Delivered by: Physician, nurse practitioner, nurse care manager, <u>and</u> social worker	costs		

			congestive heart failure (52%)	heart failure (41%)			
Luck et al [18] (also Fleischer et al [19])<br><br>Germany<br><br>RCT, 2 arms (1 intervention, 1 control)<br><br>Number of follow-ups: 1	<u>Intervention:</u> Falls risk assessment and personalized counselling<br><br><u>Control</u> No treatment	Aged 80+y, living at home, functional impairment 3* ADLs<br><br>Excluded: Cognitive impairment, need for >90min assistance per day	Baseline N = 150<br><br>Follow-up N = 118<br><br>Mean age: 85y<br><br>65% female<br><br>Ethnicity NR<br><br>Health	Baseline N = 155<br><br>Follow-up N = 112<br><br>Mean age: 85y<br><br>72% female<br><br>Ethnicity NR<br><br>Health	Behaviour(s) targeted: Taking nutritional supplements<br><br>BCTs: Adding objects to the environment, feedback on behaviour, monitoring of behaviour by others without feedback, restructuring the physical environment, social support from	*Physical functioning*<br>Falls	*Physical functioning*<br>**Evidence of potential effectiveness**

Follow-up: 18 months<br><br>Low risk of bias 4/7<br><br>No theory mentioned			conditions NR	conditions NR	intervention provider (unspecified)<br><br>Functions: Education, enablement, environmental restructuring<br><br>Setting: Home-only<br><br>Delivered by: Psychologist, sociologist or nurse scientist		
Marek et al [20] (also	<u>Intervention 1 (MD2):</u>	Aged 60+y, Medicare,	<u>Intervention 1 (MD2)</u>	Baseline N = 125	<u>Intervention 1 (MD2):</u> Behaviour targeted:	*Mental health and functioning*	<u>Intervention 1 (MD2):</u>

Marek & Antle [21])<br><br>USA<br><br>RCT, 3 arms (2 interventions, 1 control)<br>******<br><br>Number of follow-ups: 4<br><br>First follow-up: 3 months	Medication-dispensing machine<br><br><u>Intervention 2 (planner):</u><br>Medication planner<br><br><u>Control:</u><br>No treatment	impaired ability to manage medications and/or impaired cognitive functioning but able to follow directions with prompting<br><br>Excluded: Terminal diagnosis or hospice care, existing use of device for	Baseline N = 152<br><br>Follow-up N = 117<br><br>Mean age: 80y<br><br>68% female<br><br>82% White, 18% Black<br><br>Three most prevalent	Follow-up N = 116<br><br>Mean age: 78y<br><br>62% female<br><br>90% White, 10% Black<br><br>Three most prevalent health conditions: Diabetes	Medication adherence<br><br>BCTs: Adding objects to the environment, feedback on behaviour, goal setting (outcome), prompts/cues, social support from intervention provider (practical)<br><br>Functions: Enablement, environmental restructuring<br><br>Setting: Home-only	Depression<br>Cognitive function<br><br>*Physical functioning*<br>Functional performance<br><br>*Generic health and wellbeing*<br>Quality of life	*Mental health and functioning*<br>**No evidence of effectiveness**<br><br>*Physical functioning*<br>**No evidence of effectiveness**<br><br>*Generic health and wellbeing*<br>**No evidence of effectiveness**<br><br><u>Intervention 2</u>

*******<br><br>Low risk of bias 5/7<br><br>Theory mentioned: Individual and Family Self-Management Theory		medications	health conditions: Diabetes (39%), depression (20%), COPD (14%), atrial fibrillation (14%)<br><br><u>Intervention 2 (planner)</u><br><br>Baseline N = 137	(38%), depression (14%), ischemic heart disease (14%)	Delivered by: Nurse<br><br><u>Intervention 2 (planner):</u><br>Behaviour targeted: Medication adherence<br><br>BCTs: Adding objects to the environment, feedback on behaviour, goal setting (outcome), prompts/cues, social support from intervention provider (practical)		<u>(planner):</u><br>*Mental health and functioning*<br>**Evidence of potential effectiveness**<br>Less depression, better cognitive function<br><br>*Physical functioning*<br>**Evidence of potential effectiveness**

			Follow-up N = 119<br><br>Mean age: 80y<br><br>68% female<br><br>83% White, 16% Black<br><br>Three most prevalent health conditions: Diabetes (37%),		Functions: Enablement, environmental restructuring<br><br>Setting: Home-only<br><br>Delivered by: Nurse		*Generic health and wellbeing*<br>**Evidence of potential effectiveness**

			depression (28%), COPD (15%)				
Markle-Reid et al [22]<br><br>Canada<br><br>RCT, 2 arms (1 intervention, 1 control)<br><br>Number of follow-ups: 1<br><br>Follow-up: 6	<u>Intervention:</u> Health assessment, health education, coordination of community services, and use of empowerment strategies<br><br><u>Control:</u>	Aged 75+y, newly referred to and eligible for community care personal support services<br><br>Excluded: Ineligible for nursing services	Baseline N = 144<br><br>Follow-up N = 120<br><br>Modal age 75-85y (75%)<br><br>78% female<br><br>76% Canadian, 24% other	Baseline N = 144<br><br>Follow-up N = 122<br><br>Modal age: 75-85y (64%)<br><br>76% female<br><br>79% Canadian, 21% other	Behaviour targeted: Medication management<br><br>BCTs: Goal-setting (outcome), information on health consequences, monitoring of outcomes by others without feedback, social support from intervention provider (practical), social support from	*Mental health and functioning*<br>Depression<br>Mental health<br><br>*Physical functioning*<br>Functional performance<br><br>*Social functioning and wellbeing*	*Mental health and functioning*<br>**Evidence of potential effectiveness**<br>Less depression, greater mental health<br><br>*Physical functioning*<br>**Evidence of potential**

months<br><br>Low risk of bias 6/7<br><br>Theory mentioned: Model of Vulnerability	Usual home care		50% had one health disorder, 50% had two	45% had one health disorder, 55% had two	intervention provider (unspecified)<br><br>Functions: Education, enablement, training<br><br>Setting: Home-only<br><br>Delivered by: Nurse	Emotional health<br>Social functioning	**effectiveness**<br><br>*Social functioning and wellbeing*<br>**Evidence of potential effectiveness**<br>Greater emotional health
Markle-Reid et al [23]<br><br>Canada<br><br>RCT, 2 arms (1 intervention,	<u>Intervention:</u> Usual home care, plus visits from multidisciplinary team for risk and health	Aged 75+y, newly referred to and eligible for community care personal support services, at risk for falls	Baseline N = 54<br><br>Follow-up N = 49<br><br>Modal age: 75-	Baseline N = 55<br><br>Follow-up N = 43<br><br>Modal age: 75-	Behaviours targeted: Medication adherence, PA<br><br>BCTs: Goal setting (outcome), monitoring of outcomes by others	*Behavioural*<br>Nutritional status<br><br>*Health and social service use*	*Behavioural*<br>**No evidence of effectiveness**<br><br>*Health and social service use*<br>**No evidence of**

1 control)<br><br>Number of follow-ups: 1<br><br>Follow-up: 6 months<br><br>Low risk of bias 6/7<br><br>No theory mentioned	assessment, and provision of falls prevention advice<br><br><u>Control:</u> Usual home care	Excluded: N/A	85y (57%)<br><br>67% female<br><br>Ethnicity NR<br><br>Three most prevalent cardiovascular, neurological or musculoskeletal conditions: arthritis (78%), hypertension (59%), non-hip fractures	85y (51%)<br><br>77% female<br><br>Ethnicity NR<br><br>Three most prevalent cardiovascular, neurological or musculoskeletal conditions: arthritis (74%), hypertension (47%), osteoporosis	without feedback, problem solving, restructuring the physical environment, social support from intervention provider (emotional), social support from intervention provider (unspecified)<br><br>Functions: Education, environmental restructuring<br><br>Setting: Home-only	Number of acute hospital days for a fall<br><br>*Mental health and functioning*<br>Depression<br>Cognitive function<br><br>*Physical functioning*<br>Falls<br>Slips and trips<br>Gait and balance	**effectiveness**<br><br>*Mental health and functioning*<br>**No evidence of effectiveness**<br><br>*Physical functioning*<br>**No evidence of effectiveness**<br><br>*Social functioning and wellbeing*<br>**No evidence of effectiveness**

			(43%)	(47%)	Delivered by: CCAC case manager, registered nurse, occupational therapist, physiotherapist, and registered dietitian	*Social functioning and wellbeing*<br>Emotional health	
Melis et al [24] (also Melis et al [25])<br><br>Netherlands<br><br>Pseudo-cluster RCT, 2 arms (1 intervention,	<u>Intervention:</u><br>Assessment of health and development of treatment plan<br><br><u>Control:</u><br>Usual care	Aged 70+y, living at home or in retirement home, recently presented with cognitive disorders, dementia, mobility	Baseline N = 85<br><br>Follow-up N = 81<br><br>Mean age: 82y<br><br>67% female	Baseline N = 66<br><br>Follow-up N = 59<br><br>Mean age: 83y<br><br>74% female	Behaviour targeted: Dietary consumption<br><br>BCTs: Goal setting (outcome), monitoring of outcomes of behaviour by others without feedback, social support from	*Mental health and functioning*<br>Mental wellbeing<br>Dementia quality of life (negative affect)<br>Dementia quality of life	*Mental health and functioning*<br>**Evidence of potential effectiveness**<br>Enhanced mental wellbeing, reduced negative affect

1 control)<br><br>Number of follow-ups: 2<br><br>Follow-up: 3 months<br><br>Low risk of bias 7/7<br><br>No theory mentioned		disorders and falling, and/or malnutrition, with request for help related to this problem(s)<br><br>Excluded: Problem or request for help requires action within 1 week, or is only a medical diagnostic issue; proven	Ethnicity NR<br><br>Health conditions NR	Ethnicity NR<br><br>Health conditions: NR	intervention provider (unspecified)<br><br>Functions: (None identified)<br><br>Setting: Home-only<br><br>Delivered by: Geriatric specialist nurse	(positive affect)<br><br>*Physical functioning*<br>Functional performance<br>Mobility<br><br>*Social functioning and wellbeing*<br>Loneliness	*Physical functioning*<br>**Evidence of potential effectiveness**<br>Enhanced functional performance<br><br>*Social functioning and wellbeing*<br>**No evidence of effectiveness**

		moderate to severe dementia and no informal caregiver; receiving other forms of intermediate care or health care from social worker or geriatrician; on waiting list for nursing home because of problem presented; or					

		terminal illness with life expectancy <6 months					
Metzelthin et al [26] (also Metzelthin [27])<br><br>Netherlands<br><br>Cluster RCT, 2 arms (1 intervention, 1 control)	<u>Intervention:</u> Frailty and frailty risk assessment and development of personalized treatment plan<br><br><u>Control:</u> Usual care	Aged 70+y<br><br>Excluded: Terminally ill, confined to bed, or severe cognitive or psychological impairments	Baseline N = 193<br><br>Follow-up N = 171<br><br>Mean age: 77y<br><br>55% female<br><br>Ethnicity NR	Baseline N = 153<br><br>Follow-up N = 145<br><br>Mean age: 77y<br><br>61% female<br><br>Ethnicity NR	Behaviour targeted: PA<br><br>BCTs: Adding objects to the environment, feedback on outcomes of behaviour, goal setting (outcome), monitoring of outcomes of behaviour by others without feedback, restructuring physical environment, social	*Mental health and functioning*<br>Depression<br><br>*Physical functioning*<br>Functional performance<br><br>*Social functioning and wellbeing*	*Mental health and functioning*<br>**No evidence of effectiveness**<br><br>*Physical functioning*<br>**No evidence of effectiveness**<br><br>*Social functioning and wellbeing*

Number of follow-ups: 3<br><br>First follow-up: 6 months<br><br>Low risk of bias 6/7<br><br>No theory mentioned			Health conditions NR	Health conditions NR	support from intervention provider (emotional), social support from intervention provider (unspecified)<br><br>Functions: Enablement, environmental restructuring<br><br>Setting: Home-only<br><br>Delivered by: Practice nurse	Social participation	**No evidence of effectiveness**
Siu et al [28]	<u>Intervention</u>:	65+y, recent	Baseline N =	Baseline N =	Behaviour targeted:	*Behavioural*	*Behavioural*

USA<br><br>RCT, 2 arms (1 intervention, 1 control)<br><br>Number of follow-up points: 2<br><br>First follow-up: 30 days<br><br>Low risk of bias 5/7	Physical health assessment prior to hospital discharge, follow-up home visit to patient, recommendations made to patient's physician<br><br><u>Control:</u><br>Usual care	hospitalization episode, with unstable medical problems, recent functional limitations, or potentially reversible geriatric clinical problems<br><br>Excluded: Admitted from nursing homes, terminal illness	178<br><br>Follow-up N NR (total N ≤ 315)<br><br>Age range NR (≥65y)<br><br>32% male<br><br>23% Black<br><br>Three most prevalent health	176<br><br>Follow-up N NR<br>(total N ≤ 315)<br><br>Age range NR (≥65y)<br><br>48% male<br><br>15% Black<br><br>Three most prevalent health	Medication adherence<br><br>BCTs: Monitoring of outcomes of behaviour without feedback, social support from intervention provider (unspecified)<br><br>Functions: (None identified)<br><br>Setting: Home- and hospital-based<br><br>Delivered by: Nurse	Medication adherence<br><br>*Health and social service use*<br>Hospital admissions<br>Number of medications<br>Nursing home admission<br><br>*Mental health and functioning*<br>Mental health	**No evidence of effectiveness**<br><br>*Health and social service use*<br>**No evidence of effectiveness**<br><br>*Mental health and functioning*<br>**No evidence of effectiveness**<br><br>*Physical functioning*<br>**No evidence of**

No theory mentioned		with life expectancy <6 months, or expected to be hospitalized for <48 hours	conditions: Hypertension (58%), diabetes (19%), congestive heart failure (19%)	conditions: Hypertension (57%), congestive heart failure (23%), diabetes (13%)	practitioner	Role function affected by emotional problems<br><br>*Physical functioning*<br>Functional performance<br>Pain<br>Role function affected by physical problems<br>Energy/fatigue	**effectiveness**<br><br>*Social functioning and wellbeing*<br>**No evidence of effectiveness**<br><br>*Generic health and wellbeing*<br>**No evidence of effectiveness**

						Social functioning and wellbeing<br>Social functioning<br><br>*Generic health and wellbeing*<br>General health<br>Mortality<br>Health-related quality of life	
Stuck et al [29]	<u>Intervention:</u><br>Health	Aged 75+y, on health insurance	Baseline N = 148 ********	Baseline N = 296	Behaviour(s) targeted: 'Self-care'	*Behavioural*<br>Influenza	*Behavioural*<br>**Evidence of**

Switzerland<br><br>Stratified RCT, 2 arms (1 intervention, 1 control)<br><br>Number of follow-up points: 2<br><br>First follow-up: 2 years<br><br>Low risk of bias 7/7	assessments and development of treatment plan<br><br><u>Control:</u> Unclear	list<br><br>Excluded: N/A	Follow-up N = 138<br><br>Mean age: 82y<br><br>77% female<br><br>Ethnicity NR<br><br>More than 3 chronic conditions, prevalence (total intervention	Follow-up N = 278<br><br>Mean age: 82y<br><br>71% female<br><br>Ethnicity NR<br><br>More than 3 chronic conditions, prevalence (total control group): 10%	BCTs: Monitoring of outcomes of behaviour without feedback, social support from intervention provider (unspecified)<br><br>Functions: Education, enablement<br><br>Setting: Home-only<br><br>Delivered by: Public health nurse	vaccination status<br><br>*Health and social service use*<br>Hospital admissions<br>Length of hospital stay<br>Hospital care costs<br>Number of medications<br>Visits to primary care	**potential effectiveness**<br>Greater influenza vaccination<br><br>*Health and social service use*<br>**No evidence of effectiveness**<br>*Increased* number of medications<br><br>*Mental health and functioning*<br>**No evidence of effectiveness**

No theory mentioned			group): 10%			provider<br>Visits to specialist physicians<br>Home care use<br>Ambulatory care costs<br><br>*Mental health and functioning*<br>Affect<br>Cognitive function<br><br>*Physical functioning*	*Physical functioning*<br>**Evidence of potential effectiveness**<br><br>*Generic health and wellbeing*<br>**No evidence of effectiveness**

						Gait and balance<br><br>*Generic health and wellbeing*<br>General health	
Van Hout et al [30]<br><br>Netherlands<br><br>RCT, 2 arms (1 intervention, 1 control)<br><br>Number of	<u>Intervention:</u> Assessment of care needs, development of tailored care plan, and telephone monitoring<br><br><u>Control:</u>	Aged 75+y, living at home<br><br>Excluded: Terminally ill, dementia symptoms, or living in residential home	Baseline N = 331<br><br>Follow-up N = 224<br><br>Mean age: 81y<br><br>72% female	Baseline N = 320<br><br>Follow-up N = 229<br><br>Mean age: 82y<br><br>69% female	Behaviour targeted: Medication adherence<br><br>BCTs: Monitoring of outcomes by others without feedback, social support from intervention provider (unspecified)	*Health and social service use*<br>Hospital admissions<br>Acute hospital visit<br>Time to institutionalization	*Health and social service use*<br>**No evidence of effectiveness**<br><br>*Mental health and functioning*<br>**No evidence of effectiveness**

follow-ups: 2<br><br>First follow-up: 6 months<br>*******<br><br>Low risk of bias 6/7<br><br>No theory mentioned	Varied – some received no care at all, others received regular primary care physician home visits		Ethnicity NR<br><br>Three most prevalent health conditions: diabetes (50%), heart infarction (40%), hypertension (28%)	Ethnicity NR<br><br>Three most prevalent health conditions: diabetes (49%), heart infarction (37%), hypertension (29%)	Functions: (None identified)<br><br>Setting: Home-only<br><br>Delivered by: Community nurse	*Mental health and functioning*<br>Mental health<br><br>*Physical functioning*<br>Physical functioning<br>ADLs<br>IADLs<br><br>*Generic health and wellbeing*<br>Time to death	*Physical functioning*<br>**No evidence of effectiveness**<br><br>*Generic health and wellbeing*<br>**No evidence of effectiveness**
Williams et al	<u>Intervention:</u>	Aged 75+y,	Baseline N =	Baseline N =	Behaviour(s) targeted:	*Physical*	*Health and social*

[31]<br><br>UK<br><br>RCT, 2 arms (1 intervention, 1 control)<br><br>Number of follow-ups: 1<br><br>Follow-up: 1 year<br><br>Low risk of bias 5/7	Assessment of health and care needs, provision of advice<br><br><u>Control:</u> No health visits unless required	discharged from hospital in previous year<br><br>Excluded: N/A	218<br><br>Follow-up N = 176<br><br>Age NR<br><br>Gender NR<br><br>Ethnicity NR<br><br>Health conditions NR	239<br><br>Follow-up N = 188<br><br>Age NR<br><br>Gender NR<br><br>Ethnicity NR<br><br>Health conditions NR	Dietary consumption, medication use, sleeping<br><br>BCTs: Monitoring of outcomes of behaviour by others without feedback<br><br>Functions: (None identified)<br><br>Setting: Home-only<br><br>Delivered by: Health visitor assistants	*functioning*<br>Functional status<br>Disability level<br><br>*Mental health and functioning*<br>Mental status<br><br>*Health and social service use*<br>Health and social service use	*service use*<br>**No evidence of effectiveness**<br><br>*Mental health and functioning*<br>**No evidence of effectiveness**<br><br>*Physical functioning*<br>**No evidence of effectiveness**

No theory mentioned							

Abbreviations: BCT = Behaviour Change Technique. N/A = Not applicable. NR = Not reported. PA = Physical activity. RCT = Randomized controlled trial. 'Evidence of potential effectiveness' indicates significant ($p<.05$) between-group change in outcome, favorable to intervention group, in at least one outcome within the corresponding cluster. Comments have been added to the 'summary of findings' column for clarification in cases of multiple outcomes within one cluster.

Other footnotes:

* No paper used different theories to inform different intervention treatments, so theory use is described as a study characteristic.

** Relates to first follow-up point.

*** Avlund et al [1] reported sample sizes within age bands (60-69y, 70+y), not actual ages. We estimated mean age by assuming that those in the 60-69y band were all 60y, and those 70+y were 70y.

**** 'No evidence of effectiveness' denotes no between-group changes, relative to a comparator treatment (in 2-arm trials) or the control group (in 3-arm trials), in any outcomes measured within the relevant cluster.

***** Hall et al [14] included two control groups. Data were extracted for the one control group against which intervention effects were compared.

****** Marek et al [20] compared intervention 1 against intervention 2 only, and intervention 2 against control only. Effectiveness estimates for intervention 1 are thus derived from comparison against another intervention treatment, not the no-treatment control group.

******* For two papers (Marek et al [20]; van Hout et al [30]), in which changes in outcomes were reported only as trends across multiple follow-up points (Marek et al: 3, 6, 9, 12 months; van Hout et al: 6, 18 months), evidence of potential effectiveness is based on trend analyses across multiple time-points.

******** Stuck et al [29] reported outcomes at first follow-up only for a subsample of participants (i.e. those at low baseline risk for nursing home admission). Intervention and control group descriptions are based on the low-baseline-risk group where possible.

Supplemental Table 3. Risk of bias assessment

	Random sequence generation	*Allocation concealment*	*Blinding of participants and personnel*	*Blinding of outcome assessment*	*Incomplete outcome data*	*Selective reporting*	*Other sources of bias*	*Low risk score*
Avlund [1]	–	–	+	+	+	+	+	**5**
Boult [3]	+	+	+	+	+	+	+	**7**
Bouman [5]	+	**?**	+	+	+	+	+	**6**
Dalby [7]	+	–	+	+	–	+	+	**5**
Favela [8]	**?**	**?**	+	–	+	+	+	**4**
Gustafsson [9]	**?**	+	+	+	+	+	+	**6**
Hall [14]	+	**?**	+	+	+	+	+	**6**
Kono [15]	+	**?**	+	**?**	+	+	+	**5**
Levine [17]	+	**?**	+	+	+	+	+	**6**
Luck [18]	+	+	+	–	–	–	+	**4**
Marek [20]	+	**?**	+	–	+	+	+	**5**
Markle-Reid [22]	+	**?**	+	+	+	+	+	**6**

Markle-Reid [23]	+	**?**	+	+	+	+	+	**6**
Melis [24]	+	+	+	+	+	+	+	**7**
Metzelthin [26]	+	–	+	+	+	+	+	**6**
Siu [28]	+	**?**	+	+	–	+	+	**5**
Stuck [29]	+	+	+	+	+	+	+	**7**
van Hout [30]	+	**?**	+	+	+	+	+	**6**
Williams [31]	+	**?**	+	+	–	+	+	**5**

– High risk of bias

+ Low risk of bias

? Unclear risk of bias

Supplementary Table 4. Definitions and frequency of behaviour change techniques, with illustrative examples from reviewed studies

Technique	No. interventions in which BCT used	Definition *	Example and source
Action planning	2	Prompt detailed planning of performance of the behaviour (must include at least one of context, frequency, duration and intensity). Context may be environmental (physical or social) or internal (physical, emotional or cognitive)	Training participants in appropriate medication dosage, frequency and timing[8]
Adding objects to the environment	5	Add objects to the environment in order to facilitate performance of the behaviour	Providing participant with medication dispenser[20]
Discrepancy between current behaviour and goal	1	Draw attention to discrepancies between a person's current behaviour (in terms of the *form, frequency, duration, or intensity* of that behaviour) and the person's previously set	Raising and discussing differences between current behaviour and health goal[3]

Technique	No. interventions in which BCT used	Definition *	Example and source
		outcome goals, behavioural goals or action plans (goes beyond self-monitoring of behaviour)	
Feedback on behaviour	3	Monitor and provide informative or evaluative feedback on performance of the behaviour *(e.g. form, frequency, duration, intensity)*	Provide feedback on missed doses of medication[20]
Feedback on outcomes of behaviour	1	Monitor and provide feedback on the outcome of performance of the behaviour	Evaluate participants' adherence to goals and communicate adherence back to participant (unclear whether goals behavioural)[26]
Goal setting (outcome)	10	Set or agree on a goal defined in terms of a positive **outcome** of wanted behaviour (code where unclear whether goal refers to behaviour	Setting goals that meet older person's care needs (unclear whether goal specifies behaviour or outcome or

Technique	No. interventions in which BCT used	Definition *	Example and source
		or outcome of behaviour)	behaviour) [26]
Graded tasks	2	Set easy-to-perform tasks, making them increasingly difficult, but achievable, until behaviour is performed	Making a plan of incremental physical activity each week [8]
Information on health consequences	2	Provide information (e.g. written, verbal, visual) about health consequences of performing the behaviour	Informing participants of the impact of physical activity on physical fitness[9]
Instruction on how to perform behaviour	4	Advise or agree on how to perform the behaviour	Instructing participant on how to use their medication[9]
Monitoring of behaviour by others without feedback	7	Observe or record behaviour with the person's knowledge as part of a behaviour change strategy	Performing assessment of participant's physical activity[14]

Technique	No. interventions in which BCT used	Definition *	Example and source
		(code where unclear whether feedback given or not)	
Monitoring of outcomes of behaviour by others without feedback	13	Observe or record outcomes of behaviour with the person's knowledge as part of a behaviour change strategy<br>(code where unclear whether feedback given or not)	Performing assessment of participant's specific health problems, unclear whether fed back[1]
Problem solving	1	Analyze, or prompt the person to analyze, factors influencing the behaviour and generate or select strategies that include overcoming barriers and/or increasing facilitators	Using motivational interviewing to address barriers to falls prevention and promote positive changes in behaviour to reduce falls risk[23]
Prompts/cues	2	Introduce or define environmental or social stimulus with the purpose of prompting or	Provide medication dispenser or planner

Technique	No. interventions in which BCT used	Definition *	Example and source
		cueing the behaviour. The prompt or cue would normally occur at the time or place of performance	as a reminder to take medication[20]
Restructuring the physical environment	5	Change, or advise to change the **physical** environment in order to facilitate performance of the wanted behaviour or create barriers to the unwanted behaviour (other than prompts/cues, rewards and punishments)	Advising to make housing modifications to reduce fall risks[9]
Review behavioural goals	1	Review behaviour goal(s) jointly with the person and consider modifying goal(s) or behaviour change strategy in light of achievement. This may lead to re-setting the same goal, a small change in that goal or setting a new goal instead	Reassessing earlier-set behavioural goals in light of participants' progress towards them [5]

Technique	No. interventions in which BCT used	Definition *	Example and source
		of (or in addition to) the first, or no change	
Review outcome goals	3	Review outcome goal(s) jointly with the person and consider modifying goal(s) in light of achievement. This may lead to re-setting the same goal, a small change in that goal or setting a new goal instead of, or in addition to the first	Reassessing earlier-set outcome goals in light of participants' progress towards them, and proposing different goals where not achieved[8]
Self-monitoring (outcome)	1	Establish a method for the person to monitor and record the **outcome(s)** of their behaviour as part of a behaviour change strategy (Code where unclear whether monitoring behaviour or outcome)	Self-monitoring (unclear whether monitoring behaviour or outcomes)[3]
Social support from friends/family/caregivers	4	Advise on, arrange or provide social support (*from friends, family, or caregivers)* or non-	Involving family and caregivers in developing care plan (contents of plan

Technique	No. interventions in which BCT used	Definition *	Example and source
(unspecified)		contingent praise or reward for performance of the behaviour. It includes encouragement and counselling, but only when it is directed at the **behaviour.** (Code where unclear whether social support is practical or emotional)	and ways in which involved unclear)[7]
Social support from intervention provider (emotional)	3	Advise on, arrange, or provide **emotional** social support *(from those delivering intervention)* for performance of the behaviour	Home visitor advises on how to arrange to meet with other older people, to alleviate loneliness and so facilitate physical activity in the presence of others[14]
Social support from	13	Advise on, arrange, or provide **practical** help	Intervention provider providing

Technique	No. interventions in which BCT used	Definition *	Example and source
intervention provider (practical)		(*from those delivering intervention)* for performance of the behaviour	transport to facilitate attendance at physical activity classes[1]
Social support from intervention provider (unspecified)	13	Advise on, arrange or provide social support (*from those delivering intervention)* or non-contingent praise or reward for performance of the behaviour. It includes encouragement and counselling, but only when it is directed at the **behaviour.**<br><br>(Code where unclear whether social support is practical or emotional)	Intervention provider making home visits to participants[1]

Technique definitions taken verbatim from[32] (Electronic Supplementary Materials Table 3). Citations are of records reporting interventions that featured these examples, but, in instances of multiple publications arising from a single trial, not necessarily the record that best describes such intervention content.

SUPPLEMENTARY REFERENCES

** References marked with an asterisk were retrieved by the systematic search and included in the review. ** References marked with two asterisks are linked publications that were searched for additional description of interventions.*

[1] * Avlund K, Jepsen E, Vass M, *et al*. Effects of comprehensive follow-up home visits after hospitalization on functional ability and readmissions among old patients. A randomized controlled study. *Scan J Occup Ther* 2002;**9**: 17-22.

[2] ** Vass M, Avlund K, Hendriksen C, *et al.* Preventive home visits to older people in Denmark: Methodology of a randomized controlled study. *Aging Clin Exp Res* 2002; **14:** 509-515.

[3] * Boult C, Leff B, Boyd CM, *et al.* A matched-pair cluster-randomized trial of guided care for high-risk older patients. *J Gen Intern Med* 2013;**28**: 612-621.

[4] ** Boyd CM, Boult C, Shadmi E, *et al.* Guided care for multimorbid older adults. *The Gerontologist* 2007;**47**: 697-704.

[5] * Bouman A, van Rossum E, Ambergen T, *et al*. Effects of a home visiting program for older people with poor health status: a randomized, clinical trial in The Netherlands. *J Am Geriatr Soc* 2008;**56**: 397-404.

[6] ** Nicolaides-Bouman A, van Rossum E, Kempen GIJM, *et al.* Effects of home visits by home nurses to elderly people with health problems: design of a randomized clinical trial in the Netherlands. *BMC Health Serv Res* 2004;**4**:35.

[7] * Dalby DM, Sellors JW, Fraser FD, *et al*. Effect of preventive home visits by a nurse on the outcomes of frail elderly people in the community: a randomized controlled trial. *Can Med Ass J* 2000;**162**: 497-500.

[8] * Favela J, Castro LA, Franco-Marina F, *et al.* Nurse home visits with or without alert buttons versus usual care in the frail elderly: a randomized controlled trial. *Clin Interv Aging* 2013;**8**: 85-95.

[9] * Gustafsson S, Edberg A-K, Johansson B, *et al*. Multi-component health promotion and disease prevention for community dwelling frail elderly persons: a systematic review. *Eur J Ageing* 2009;**6**: 315-329.

[10] ** Behm L, Dahlin-Ivanoff S, Zidén L. Preventive home visits and health – experiences among very old people. *BMC Pub Health* 2013;**13**: 378.

[11] ** Behm L, Wilhelmson K, Falk K, *et al.* Positive health outcomes following health-promoting and disease-preventive interventions for independent very old persons: Long-term results of the three-armed RCT Elderly Persons in the Risk Zone. *Arch Gerontol Geriatr* 2014;**58**: 376-383.

[12] ** Behm L, Zidén L, Dunér A, *et al.* Multi-professional and multi-dimensional group education – a key to action in elderly persons. *Disab Rehab* 2013; **35:** 427-435.

[13] ** Dahlin-Ivanoff S, Gosman—Hedström G, Edberg A-K, *et al*. Elderly persons in the risk zone. Design of a multidimensional, health-promoting, randomised three-armed controlled trial for “prefrail” people of 80+ years living at home. *BMC Geriatrics* 2010;**10**: 27.

[14] * Hall N, De Beck P, Johnson D, *et al*. Randomized trial of a health promotion program for frail elders. *Can J Aging* 1992;**11**: 72-91.

[15] * Kono A, Kanaya Y, Fujita T, *et al*. Effects of a preventive home visit program in ambulatory frail older people: A randomized controlled trial. *J Gerontol A Biol Sci Med Sci* 2012;**67A**: 302-309.

[16] ** Kono A, Fujita T, Tsumura C, *et al*. Preventive home visit model targeted to specific care needs of ambulatory frail elders: preliminary report of a randomized trial design. *Aging Clin Exp Res* 2009;**21**: 167-173.

[17] * Levine S, Steinman BA, Attaway K, *et al*. Home care program for patients at high risk of hospitalization. *Am J Manag Care* 2012;**18**: e269-e276.

[18] * Luck T, Motzek T, Luppa M, *et al*. Effectiveness of preventive home visits in reducing the risk of falls in old age: a randomized controlled trial. *Clin Interv Aging* 2013;**8**: 697-702.

[19] ** Fleischer S, Roling G, Beutner K, *et al*. Growing old at home – A randomized controlled trial to investigate the effectiveness and cost-effectiveness of preventive home visits to reduce nursing home admissions: study protocol. *BMC Pub Health* 2008;**8**: 185.

[20] * Marek KD, Stetzer F, Ryan PA, *et al.* Nurse care coordination and technology effects on health status of frail older adults via enhanced self-management of medication: Randomized clinical trial to test efficacy. *Nurs Res* 2013;**62**: 269-278.

[21] ** Marek KD, Antle L. Medication management of the community-dwelling older adult. In RG Hughes (Ed.), *Patient safety and quality: An evidence-based handbook for nurses* (Vol. 1). Rockville: Agency for Healthcare Research and Quality.

[22] * Markle-Reid M, Weir R, Browne G, *et al.* Health promotion for frail older home care clients. *J Adv Nurs* 2006;**54:** 381-393.

[23] * Markle-Reid M, Browne G, Gafni A, *et al.* The effects and costs of a multifactorial and interdisciplinary team approach to falls prevention for older home care clients 'at risk' for falling: a randomized controlled trial. *Can J Aging* 2010;**29**: 139-161.

[24] * Melis RJF, van Eijken MIJ, Teerenstra S, *et al.* A randomized study of a multidisciplinary program to intervene on geriatric syndroms in vulnerable older people who live at home (Dutch EASYcare Study). *J Gerontol Med Sci* 2008; **63A**: 283-290.

[25] ** Melis RJF, van Eijken MIJ, Borm GF, *et al.* The design of the Dutch EASYcare study: a randomized controlled trial on the effectiveness of a problem-based community intervention model for frail elderly people. *BMC Health Serv Res* 2005;**5**: 65.

[26] * Metzelthin SF, Van Rossum E, De Witte LP, *et al.* Effectiveness of interdisciplinary primary care approach to reduce disability in community dwelling frail older people: Cluster randomised controlled trial. *BMJ* 2013;**347**.

[27] ** Metzelthin SF. An interdisciplinary primary care approach for frail older people: Feasibility, effects and costs. Unpublished doctoral thesis, Maastricht University.

[28] * Siu AL, Kravitz RL, Keeler E, *et al*. Postdischarge geriatric assessment of hospitalized frail elderly patients. *Arch Intern Med* 1996;**156**: 76-81.

[29] * Stuck AE, Minder CE, Peter-Wüest I, *et al.* A randomized trial of in-home visits for disability prevention in community-dwelling older people at low and high risk for nursing home admission. *Arch Intern Med* 2000;**160**: 977-986.

[30] * van Hout HPJ, Jansen APD, van Marwijk HWJ, *et al*. Prevention of adverse health trajectories in a vulnerable elderly population through nurse home visits: a randomized controlled trial [ISRCTN05358495]. *J Gerontol A Biol Sci Med Sci* 2010;**65**: 734-742.

[31] * Williams EI, Greenwell J, Groom LM. The care of people over 75 years old after discharge from hospital: An evaluation of timetabled visiting by Health Visitor Assistants. *J Pub Health Med* 1992;**14**: 138-144.

[32] Michie S, Richardson M, Johnston M, et al. The behaviour change technique taxonomy (v1) of 93 hierarchically clustered techniques: Building an international consensus for the reporting of behaviour change interventions. *Ann Behav Med* 2013;46: 81-95.
